# Exploring neighborhoods in large metagenome assembly graphs using spacegraphcats reveals hidden sequence diversity

**DOI:** 10.1186/s13059-020-02066-4

**Published:** 2020-07-06

**Authors:** C. Titus Brown, Dominik Moritz, Michael P. O’Brien, Felix Reidl, Taylor Reiter, Blair D. Sullivan

**Affiliations:** 1 Department of Population Health and Reproduction, University of California Davis, Davis, USA; 2 Paul G. Allen School of Computer Science and Engineering, University of Washington, Seattle, USA; 3 Department of Computer Science, NC State University, Raleigh, USA

**Keywords:** Metagenomics, Sequence assembly, Strain variation, Bounded expansion, Dominating set

## Abstract

Genomes computationally inferred from large metagenomic data sets are often incomplete and may be missing functionally important content and strain variation. We introduce an information retrieval system for large metagenomic data sets that exploits the sparsity of DNA assembly graphs to efficiently extract subgraphs surrounding an inferred genome. We apply this system to recover missing content from genome bins and show that substantial genomic sequence variation is present in a real metagenome. Our software implementation is available at https://github.com/spacegraphcats/spacegraphcatsunder the 3-Clause BSD License.

## Results

### Dominating sets enable efficient neighborhood queries in large assembly graphs

**Fig. 1:**
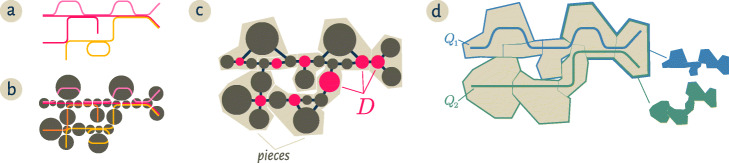
Starting from a collection of genomic sequences (**a**), we form an assembly graph where nodes represent distinct linear subsequences (**b**). In this assembly graph, known as a *compact De Bruijn graph* [4], nodes may represent many *k*-mers. The original genomic sequences correspond to walks in the graph, and shared nodes between the walks represent shared subsequences. **c** We then identify a subset of nodes *D* called a *dominating set* so that every node in the assembly graph is at distance at most one from some member of *D* (marked pink). We further partition the graph into *pieces* by assigning every node to exactly one of the closest members of *D* (beige regions in **c** and **d**). For a genomic query *Q*, the *neighborhood* of *Q* in this graph is the union of all pieces which share at least one *k*-mer with the query. The colorful subsets of the pieces in **d** correspond to the neighborhoods of the queries *Q*_1_,*Q*_2_

We designed and implemented [23] a set of algorithms for efficiently finding content in a metagenome that is close to a query as measured by distance in a compact De Bruijn graph (cDBG) representation of the sequencing data (Fig. 1). To accomplish this, we organize the cDBG into *pieces* around a set of *dominators* that are collectively close to all vertices. In this context, the *neighborhood* of a query is the union of all pieces it overlaps; to enable efficient search, we build an invertible index of the pieces.

We compute dominators so that the minimum distance from every vertex in the cDBG to some dominator is at most *r* (an *r-dominating set*) using Algorithm 1, which is based on the linear-time approximation algorithm given by Dvořák and Reidl [24]. Although finding a minimum *r*-dominating set is NP-hard [25–27] and an approximation factor below log*n* is probably impossible [26] in general graphs, our approach guarantees constant-factor approximations in linear running time by exploiting the fact that (compact) De Bruijn graphs have *bounded expansion*, a special type of sparsity [28]. Algorithm 1 first annotates the graph to determine the distances between all pairs of vertices at distance at most *r* (lines 1–3) and then uses these distances to ensure each vertex is close to a dominator. The core of the efficient distance computation is based on *distance-truncated transitive fraternal* (*dtf*) *augmentations* [24] which produce a directed graph $\overrightarrow {G}_{r}$ in which each arc *uv* is labeled with *ω*(*uv*), the distance from *u* to *v* in the original cDBG *G*.

Importantly, our implementation enhances the algorithm in [24] by computing only *r*−1 rounds of dtf augmentations instead of 2*r*−1. Since augmentation is the computationally most expensive part of the pipeline and the running time depends non-linearly on the number of rounds, this vastly improves this algorithm’s scalability. To maintain approximation guarantees on the dominating set size with fewer augmentations, we introduce a threshold parameter domThreshold(*r*) which affects the constant factor in our worst-case bound. We selected a threshold (see Additional file 1) that produces *r*-dominating sets of comparable size to those computed by the algorithm in [24]. Additionally, we found that processing vertices using a minimum in-degree ordering (line ??) was superior to other common orders (e.g., arbitrary, min/max total degree, max in-degree).


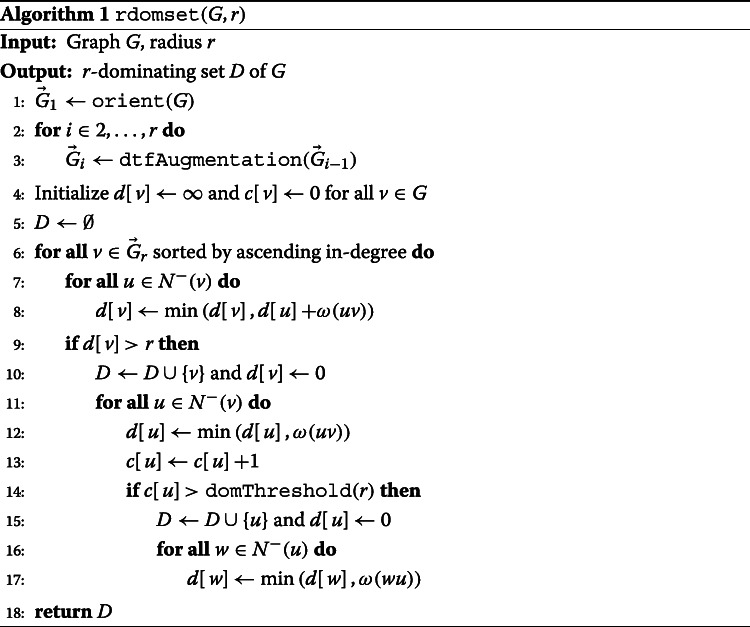


After computing an *r*-dominating set *D* of *G* with Algorithm 1, Algorithm 2 partitions the vertices of *G* into pieces so that each piece *P*[*v*] contains a connected set of vertices for which *v* is a closest member of *D* (Fig. 1). Finally, we use minimal perfect hashing (mphfIndex) [29] to compute an invertible index^1^ between pieces and their sequence content in the metagenome.

One feature of this approach is that the dominating set and index only need to be computed once for a given metagenome, independent of the number and content of anticipated queries. Queries can then be performed using Algorithm 3 in time that scales linearly with the size of their *neighborhood*—all genomic content assigned to pieces that contain part of the query.

Our implementations of these algorithms in spacegraphcats can be run on metagenomic data with millions of cDBG nodes (Table 1); indexing takes under an hour, enabling queries to complete in seconds to minutes (Table 2). See Additional file 1, Appendix D for full benchmarking (including cDBG construction). This provides us with a tool to systematically investigate assembly graph neighborhoods.

**Table 1:** Number of cDBG nodes |*V*|, edge density of cDBG |*E*|/|*V*|, size of 1-dominating set |*D*|, average query size (*k*-mers) $\overline {|Q|}$, and average number of pieces in query neighborhood $\overline {|\mathcal {P} \cap \mathcal {N}_{Q}|}$

Data set	|*V*|	|*E*|/ |*V*|	|*D*|	$\overline {|Q|}$	$\overline {|\mathcal {P} \cap \mathcal {N}_{Q}|}$
podarV	916,041	2.2	542,350	1,475,892	4106
HuSB1	13,852,950	2.6	6,724,505	1,112,516	106,091

Queries are the 51 genomes and 23 genome bins fully present in podarV and HuSB1, respectively

**Table 2:** Time and memory usage of spacegraphcats for Algorithms 1–3 on representative metagenome data

Data set	Algorithm	Time (s)	Memory (MB)
podarV	rdomset	78.1	4304
	indexPieces	359.9	14,108
	search	14.9	3463
HuSB1	rdomset	1181.1	60,238
	indexPieces	859.3	40,713
	search	67.9	15,228

The times for Algorithm 3 are averaged over all queries (see Table 1). Statistics reported for Algorithm 2 exclude lines 1–2 of pseudocode. Times are rounded to the nearest tenth of a second; memory is rounded to the nearest megabyte


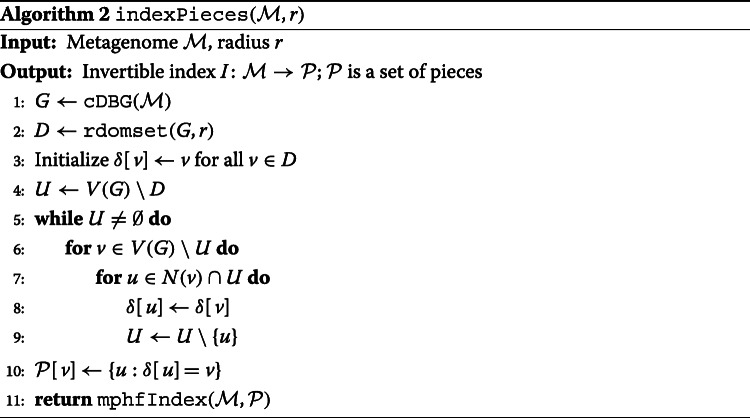



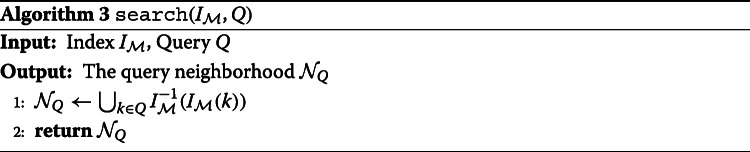


### Neighborhood queries enable recovery of relevant unknown genomic content

We first measured how well an inexact query can recover a target genome from a metagenome. For a benchmark data set, we used the podarV data set [30]. This is a “mock” metagenome containing genomes from 65 strains and species of bacteria and archaea, each grown independently and rendered into DNA, then combined and sequenced as a metagenome. This metagenome is a commonly used benchmark for assembly [12, 31–33].

**Fig. 2:**
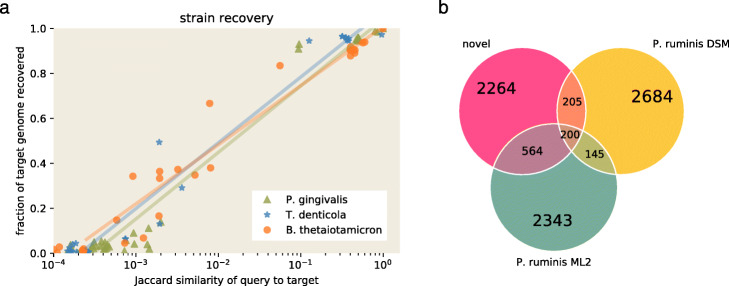
Neighborhood queries enable recovery of relevant genomic content. **a** Left panel: recovery of each of three target genomes from podarV using queries at a variety of Jaccard distances from the target. Recovery is calculated as containment of target genome in query neighborhood. The solid lines represent logarithmic best-fit curves to the points. **b** Right panel: recovery of novel *Proteiniclasticum* content from podarV. Nucleotide *k*-mers from two of the three known *P. ruminis* genomes overlapped approximately a megabase of sequence in the query neighborhood, which also contained approximately 2.3 Mbp of unknown sequence; the third known genome, *P. ruminis CGMCC*, was omitted from the figure as it is 99.7% similar to *P. ruminis DSM*. Numbers are in thousands of *k*-mers, estimated via sourmash

To evaluate the effectiveness of neighborhood query at recovering strain variants, we chose three target genomes from podarV—*Porphyromonas gingivalis ATCC 33277*, *Treponema denticola ATCC 35405*, and *Bacteroides thetaiotaomicron VPI-5482*—that have many taxonomically close relatives in GenBank. We then used these relatives to query the podarV mixture and measure the recovery of the target genome. The results, in Fig. 2a, show that graph neighborhood query can recover 35% or more of some target genomes starting from a relative with Jaccard similarity as low as 1%: even a small number of shared *k*-mers sufficed to define a much larger neighborhood that contains related genomes.

We next applied neighborhood query to retrieve an unknown genome from podarV. Several papers have noted the presence of unexpected sequence in the assemblies of this data, and Awad et al. identify at least two species that differ from the intended mock metagenome contents [12, 31]. One species variant has partial matches to several different *Fusobacterium nucleatum* genomes, while the other incompletely matches to three strains of *Proteiniclasticum ruminis*.

The complete genomes of these two variants are not in public databases and, for the *Proteiniclasticum* variant, cannot be entirely recovered with existing approaches: when we assemble the reads that share *k*-mers with the available genomes, a marker-based analysis with CheckM estimates that 98.8% of the *Fusobacterium* variant is recovered, while only 72.96% of the *Proteiniclasticum* variant is recovered. We therefore tried using neighborhood queries to expand our knowledge of the *Proteiniclasticum* variant.

We performed a neighborhood query into podarV with all three known *Proteiniclasticum* genomes from GenBank. We then extracted the reads overlapping this neighborhood and assembled them with MEGAHIT. The retrieved genome neighborhood for *Proteiniclasticum* contains 2264K novel *k*-mers (Fig. 2b). The reads from the query neighborhood assembled into a 3.1-Mbp genome bin. The assembly is estimated by CheckM to be 100% complete, with 10.3% contamination. The mean amino acid identity between *P. ruminis ML2* and the neighborhood assembly is above 95%, suggesting that this is indeed the genome of the *Proteiniclasticum* variant and that neighborhood query retrieves a full draft genome sequence (see Additional file 1, Appendix G).

### Query neighborhoods in a real metagenome do not always assemble well

Real metagenomes may differ substantially from mock metagenomes in size, complexity, and content. In particular, real metagenomes may contain a complex mixture of species and strain variants [34] and the performance of assembly and binning algorithms on these data sets is challenging to evaluate in the absence of ground truth. One recent comparison of single-cell genomes and metagenome-assembled genomes in an ocean environment found that up to 40% of single-cell genome content may be missing in metagenome-assembled genomes [15].

**Fig. 3:**
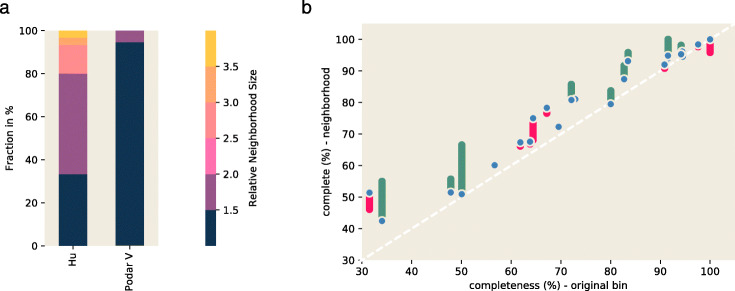
Query neighborhoods in HuSB1 metagenome are large and contain additional marker genes. **a** Left panel: neighborhood sizes are larger in HuSB1 than in podarV. Here, we queried podarV and HuSB1 using each of 51 and 23 genomes fully present in the respective data sets and measured the relative size of its neighborhood—a size of 1 indicates that no additional sequence is present in the neighborhood, while a size of 2 indicates that the retrieved neighborhood is twice the size of the query genome. **b** Right panel: query neighborhoods are estimated to be more complete than the original genome bins. We queried HuSB1 using each of 23 genomes binned from SB1 and assembled the resulting neighborhoods using MEGAHIT and Plass. The blue points represent completeness estimates of MEGAHIT-assembled neighborhoods, while green and pink bars represent the additional or missing content in the Plass assemblies, respectively

We first ask whether genome query neighborhood sizes in a real metagenome differ from mock metagenomes. We examined genomes inferred from the SB1 sample from the Hu et al. study, in which 6 metagenomic samples taken from various types of oil reservoirs were sequenced, assembled, binned, and computationally analyzed for biochemical function [35]. Examining the 23 binned genomes in GenBank originating from the SB1 sample, we compared the HuSB1 neighborhood size distribution with the podarV data set (Fig. 3a). We saw that more genome bins in HuSB1 have 1.5 × or larger query neighborhoods than do the genomes in podarV. This suggests the presence of considerably more local neighborhood content in the real metagenome than in the mock metagenome.

We next investigated metagenomic content in the query neighborhoods. As with the unknown variants in podarV, we used CheckM to estimate genome bin completeness. The estimated bin completeness for many of the query genomes is low (Additional file 1, Appendix I). To see if the query neighborhoods contain missing marker genes, we assembled reads from the query neighborhoods using MEGAHIT and found this improved the completion metrics (Fig. 3b).

Investigating further, we found that the query neighborhood assemblies contain only between 4 and 56% of the neighborhood *k*-mer content (Additional file 1, Appendix J), suggesting that MEGAHIT is not including many of the reads in the assembly of the query neighborhoods. This could result from high error rates and/or high strain variation in the underlying reads [11, 12].

To attempt the recovery of more gene content from the assemblies, we turned to the Plass amino acid assembler [36]. Plass implements an overlap-based amino acid assembly approach that is considerably more sensitive than nucleotide assemblers and could be more robust to errors and strain variation [37].

When we applied Plass to the reads from the query neighborhoods, we saw a further increase in neighborhood completeness (Fig. 3b). This suggests that the genome bin query neighborhoods contain real genes that are accessible to the Plass amino acid assembler. We note that these are unlikely to be false positives, since CheckM uses a highly specific Hidden Markov Model (HMM)-based approach to detecting marker genes [38].

### Some query neighborhoods contain substantial strain variation

If strain variation is contributing to poor nucleotide assembly of marker genes in the query neigborhoods, then Plass should assemble these variants into similar amino acid sequences. Strain variation for unknown genes can be difficult to study due to lack of ground truth, but highly conserved proteins should be readily identifiable.

The *gyrA* gene encodes an essential DNA topoisomerase that participates in DNA supercoiling and was used by [35] as a phylogenetic marker. In the GenBank bins, we found that 15 of the 23 bins contain at least one gyrA sequence (with 18 *gyrA* genes total). We therefore used *gyrA* for an initial analysis of the Plass-assembled neighborhood content for all 23 bins. To avoid confounding effects of random sequencing error in the analysis and increase specificity at the cost of sensitivity, we focused only on high-abundance data: we truncated all reads in the query neighborhoods at any *k*-mer that appears fewer than five times, and ran Plass on these abundance-trimmed reads from each neighborhood. We then searched the gene assemblies with a gyrA-derived HMM, aligned all high-scoring matches, and calculated a pairwise similarity matrix from the resulting alignment.

**Fig. 4:**
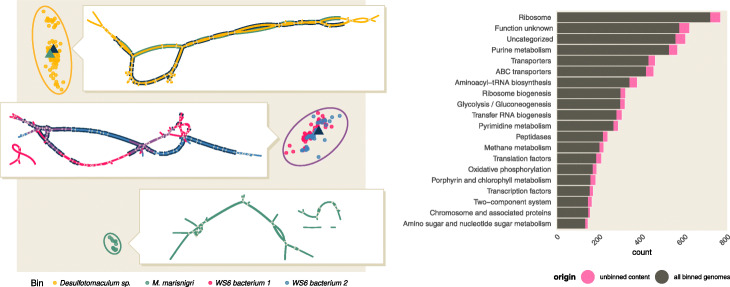
Query neighborhoods in HuSB1 contain sequence variants and new genes. **a** Left panel: gyrA has substantial minor sequence variation in several query neighborhoods. In this multidimensional scaling plot, each point represents a distinct gyrA sequence from the Plass assemblies of four representative query neighborhoods, colored by query binned genome. The triangles represent gyrA sequences originating from the query binned genome, if any are present. The inlays are visualizations of assembly graphs of reads that contain *gyrA* sequence in each neighborhood. Unitigs are colored by their cluster of origin; matches to *gyrA* sequences from the bin are highlighted using color from relevant triangle. **b** Right panel: genome neighborhoods re-associate annotated functionality to binned genomes. For each of 23 genome bins originating from HuSB1, we found the unbinned content by removing all orthologs found in the binned genomes in [39] and by counting distinct ortholog annotations once. Functional content is distributed throughout pathways present in the binned genomes and increases functionality associated with binned genomes by approximately 13%

When we examine all of the high-scoring gyrA protein matches in the hard-trimmed data, we see considerable sequence variation in some query neighborhoods (Fig. 4a). Much of this variation is present in fragmented Plass assemblies; when the underlying nucleotide sequences are retrieved and used to construct a compact De Bruijn graph, the variation is visible as spurs off of a few longer paths (insets in Fig. 4a). When we count the number of well-supported amino acid variants in isolated positions (i.e., ignoring linkage between variants), we see that ten of the 23 neighborhoods have an increased number of gyrA genes, with four neighborhoods gaining a gyrA where none exists in the bin (Additional file 1, Appendix L; see lowest inset in Fig. 4a for one example). Only one neighborhood, *M. bacterium*, loses its gyrA genes due to the stringent *k*-mer abundance trimming. Collectively, the use of the Plass assembler on genome neighborhoods substantially increases the number of gyrA sequences associated with bins.

We see this same pattern for many genes, including *alaS*, *gyrB*, *rpb2* domain 6, *recA*, *rplB*, and *rpsC* (Additional file 1, Appendix M). This shows that multiple variants of those proteins are present within at least some of the neighborhoods and implies the presence of underlying nucleotide strain variation. This strain variation may be one reason that nucleotide assembly performs poorly: on average, only 19.6% of Plass-assembled proteins are found within the nucleotide assemblies.

### Query neighborhoods assembled with Plass contain additional functional content

In addition to capturing variants close to sequences in the bins, we identify many novel genes in the query neighborhoods. We used KEGG to annotate the Plass-assembled amino acid sequences and then removed any annotations already present for genes in the genome bin. We also ignored homolog abundance such that each homolog is counted only once per neighborhood.

Novel functional content is distributed throughout pathways present in the genome bins and increases functionality associated with binned genomes by approximately 13% (Fig. 4b). This includes orthologs in biologically relevant pathways such as methane metabolism, which are important for biogeochemical cycling in oil reservoirs [35].

Genes in these neighborhoods contain important metabolic functionality expanding the pathways already identified in [35]. We find 40 unique orthologs involved in nitrogen fixation across eight neighborhoods, four of which had no ortholog in the bin. Importantly, we find the ratio of observed orthologs approximately matches that noted in [35], where two thirds of nitrogen fixation functionality is attributable to archaea (29 of 40 orthologs). This is in contrast to most ecological systems where bacteria are the dominant nitrogen fixers [35].

## Discussion

### Efficient graph algorithms provide novel tools for investigating graph neighborhoods

Recent work has shown that incorporating the structure of the assembly graph into the analysis of metagenome data can provide a more complete picture of gene content [21, 22]. While this has provided evidence that it is useful to analyze sequences that have small graph distance from a query (are in a “neighborhood”), this approach has not been widely adopted. Naïvely, local expansion around many queries in the assembly graph does not scale to these types of analyses due to the overhead associated with searching in a massive graph. The neighborhood index structure described in this work overcomes this computational obstacle and enables rapid exploration of sequence data that is local to a query.

Because a partition into pieces provides an implicit data reduction (the cDBG edge relationships are subsumed by piece membership), the query-independent nature of the index allows many queries to be processed quickly without loading the entire graph into memory. Our approach consequently provides a data exploration framework not otherwise available.

Exploiting the structural sparsity of cDBGs is a crucial component of our algorithms. First, it is necessary to use graph structure to obtain a guarantee that Algorithm 2 finds a small number of pieces since the size of a minimum *r*-dominating set cannot be approximated better than a factor of log*n* in general graphs^2^ unless NP⊆DTIME(*n*^*O*(log log*n*)^) [26]. Without such a guarantee, we cannot be sure that we are achieving significant data reduction by grouping cDBG vertices into pieces. Being able to do this in linear time also ensures that indexing and querying can scale to very large data sets. Furthermore, because we utilize a broad structural characterization (bounded expansion) of cDBGs rather than a highly specialized aspect, our methods enable neighborhood-based information retrieval in any domain whose graphs exhibit bounded expansion structure; examples include some infrastructure, social, and communication networks [24, 40, 41].

### Neighborhood queries extend genome bins

In both the podarV and HuSB1 metagenomes, neighborhood queries were able to identify additional content likely belonging to query genomes. In the podarV mock metagenome, we retrieved a potentially complete genome for an unknown strain based on partial matches to known genomes. In the HuSB1 metagenome, we increased the estimated completeness of most genome bins—in some cases substantially, e.g., in the case of *P_bacterium 34_609*, we added an estimated 20.9% to the genome bin. In both cases, we rely solely on the structure of the assembly graph to expand the genome bins. We do not make use of sequence composition, contig abundance, or phylogenetic marker genes in our search. Thus graph proximity provides an orthogonal set of information for genome-resolved metagenomics that could be used to improve current binning techniques.

### Query neighborhoods from real metagenomes contain substantial strain variation that may block assembly

Previous work suggests that metagenome assembly and binning approaches are fragile to strain variation [11, 12]. This may prevent the characterization of some genomes from metagenomes. The extent of this problem is unknown, although the majority of approaches to genome-resolved metagenomics rely on assembly and thus could be affected.

In this work, we find that some of the sequence missing from genome bins can be retrieved using neighborhood queries. For HuSB1, some genome bins are missing as many as 68.5% of marker genes from the original bins, with more than half of the 22 bins missing 20% or more; this accords well with evidence from a recent comparison of single-cell genomes and metagenome-assembled genomes [15], in which it was found that metagenome-assembled genomes were often missing 20 to 40% of single-cell genomic sequence. Neighborhood query followed by amino acid assembly recovers additional content for all but two of the genome bins; this is likely an underestimate, since Plass may also be failing to assemble some content.

When we bioinformatically analyze the function of the expanded genome content from neighborhood queries, our results are consistent with the previous metabolic analyses by [35] and extend the set of available genes by 13%. This suggests that current approaches to genome binning are specific and that the main question is sensitivity, which agrees with a more direct measurement of lost content [15].

### Neighborhood queries enable a genome-targeted workflow to recover strain variation

The spacegraphcats analysis workflow described above starts with genome bins. The genome bins are used as a query into the metagenome assembly graph, following which we extract reads from the query neighborhood. We assemble these reads with the Plass amino acid assembler and then analyze the assembly for gene content. We show that the Plass assembly contains strain-level heterogeneity at the amino acid level and that this heterogeneity is supported by underlying nucleotide diversity. Even with stringent error trimming on the underlying reads, we identify at least thirteen novel gyrA sequences in ten genome neighborhoods.

Of note, this workflow explicitly associates the Plass-assembled proteins with specific genome bins, as opposed to a whole-metagenome Plass assembly which recovers protein sequence from the entire metagenome but does not link those proteins to specific genomes. The binning-based workflow connects the increased sensitivity of Plass assembly to the full suite of tools available for genome-resolved metagenome analysis, including phylogenomic and metabolic analysis. However, spacegraphcats does not separate regions of the graph shared in multiple query neighborhoods; existing strain recovery approaches such as DESMAN or MSPminer could be used for this purpose [16, 19].

One future step could be to characterize unbinned genomic content from metagenomes by looking at Plass-assembled marker genes in the metagenome that do not belong to any bin’s query neighborhood. This would provide an estimate of the extent of metagenome content remaining unbinned.

## Conclusions

The neighborhood query approach described in this work provides an alternative window into metagenome content associated with binned genomes. We extend previous work showing that assembly-based methods are fragile to strain variation, and provide an alternative workflow that substantially broadens our ability to characterize metagenome content. This first investigation focuses on only two data sets, one mock and one real, but the neighborhood indexing approach is broadly applicable to all shotgun metagenomes.

In this initial investigation of neighborhood indexing, we have focused on using neighborhood queries with a genome bin. We recognize that this approach is of limited use in regions where no genome bin is available; spacegraphcats is flexible and performant enough to support alternative approaches such as querying with *k*-mers belonging to genes of interest.

Potential applications of spacegraphcats in metagenomics include developing metrics for genome binning quality, analyzing pangenome neighborhood structure, exploring *r*-dominating sets for *r*>1, extending analyses to colored De Bruijn graphs, and investigating de novo extraction of genomes based on neighborhood content. We could also apply spacegraphcats to analyze the neighborhood structure of assembly graphs overlayed with physical contact information (from, for example, HiC), which could yield new applications in both metagenomics and genomics [42, 43].

More generally, the graph indexing approach developed here may be applicable well beyond metagenomes and sequence analysis. The exploitation of bounded expansion to efficiently compute *r*-dominating sets on large graphs makes this technique applicable to a broad array of problems.

## Materials and methods

### Data

We use two data sets: SRR606249 from podarV [44] and SRR1976948 (sample SB1) from hu [39]. Each data set was first preprocessed to remove low-abundance *k*-mers as in [45], using trim-low-abund.py from khmer v2.1.2 [46] with the parameters -C 3 -Z 18 -M 20e9 -V -k 31. We build compact De Bruijn graphs using BCALM v2.2.0 [47]. Stringent read trimming at low-abundance *k*-mers was done with trim-low-abund.py from khmer, with the parameters -C 5 -M 20e9 -k 31.

### Benchmarking

We measured time and memory usage for Algorithms 1–3 by executing the following targets in the spacegraphcatsconf/Snakefile: catlas.csv for rdomset, contigs.fa.gz.mphf for indexPieces, and search for search. We report wall time and maximum resident set size, running under Ubuntu 18.04 on an NSF Jetstream virtual machine with 10 cores and 30 GB of RAM [48, 49]. To measure maximum resident set size, we used the memusg script (Jaeho Shin, https://gist.github.com/netj/526585).

### Graph denoising

For each data set, we built a compact De Bruijn graph (cDBG) for *k*=31 by computing the set of unitigs with BCALM [50] and removing all vertices of degree one with a mean *k*-mer abundance of 1.1 or less. After the removal of these vertices, we then contracted any newly revealed degree-two paths.

### Neighborhood indexing and search

We used spacegraphcats to build an *r*-dominating set for each denoised cDBG and index it. We then performed neighborhood queries with spacegraphcats, which produces a set of cDBG nodes and reads that contributed to them. The full list of query genomes for the *Proteiniclasticum* query is available in Additional file 1, Appendix F, and the NCBI accessions for the *P. gingivalis*, *T. denticola*, and *B. thetaiotamicron* queries are in the directory pipeline-base of the paper repository, files strain-gingivalis.txt, strain-denticola.txt, and strain-bacteroides.txt, respectively.

### Search results analysis

Query neighborhood size, Jaccard containment, and Jaccard similarity were estimated using modulo hash signatures with a *k*-mer size of 31 and a scaled factor of 1000, as implemented in sourmash v2.0a9 [51].

### Assembly and genome bin analysis

We assembled reads using MEGAHIT v1.1.3 [31] and Plass v2-c7e35 [36], treating the reads as single-ended. Bin completeness was estimated with CheckM 1.0.11, with the –reduced_tree argument [38]. Amino acid identity between bins and genomes was calculated using CompareM commit 7cd51276 (https://github.com/dparks1134/CompareM).

### Gene targeted analysis

Analysis of specific genes was done with HMMER v3.2.1 [52]. Plass amino acid assemblies were queried with HMMER hmmscan using the PFAM domains listed in Additional file 1, Table S7, using a threshold score of 100 [53]. Matching sequences were then extracted from the assemblies for further analysis. To overcome problems associated with comparing non-overlapping sequence fragments, only sequences that overlapped 125 of the most-overlapped 200 residues of the PFAM domain were retained (all sequences shared a minimum overlap of 50 residues with all other sequences). These sequences were aligned with MAFFT v7.407 with the –auto argument [54]. Pairwise similarities were calculated using HMMER where the final value represented the number of identical amino acids in the alignment divided by the number of overlapping residues between the sequences. Pairwise distances were visualized using a multidimensional scaling calculated in R using the cmdscale function. To visualize the assembly graph structure underlying these amino acid assemblies, we used paladin v1.3.1 to map abundance-trimmed reads back to the Plass amino acid assembly, with -f 125 to set the minimum ORF length accepted [55]. We extracted the reads that mapped to the gene of interest, created an assembly graph using BCALM v2.2.0 [50], and visualized the graph using Bandage v0.8.1 [56]. We colored nucleotide sequences originating from the bins using the BLAST feature in Bandage.

### KEGG analysis

We annotated the Plass assemblies using Kyoto Encyclopedia of Genes GhostKOALA v2.0 [57]. To assign KEGG ortholog function, we used methods implemented at https://github.com/edgraham/GhostKoalaParser release 1.1.

## Supplementary information

**Additional file 1** Contains supplementary text, figures, and tables.

**Additional file 2** Contains the review history.

## Footnotes

1 An invertible function that defines both an index and the corresponding inverted index

2 That is, graphs about which we make no structural assumptions.
