# Supplementary material for: Exploring neighborhoods in large metagenome assembly graphs using spacegraphcats reveals hidden sequence diversity
Source: Genome Biol. 2020 Jul 6;21:164. doi: 10.1186/s13059-020-02066-4 (PMC7336657; doi:10.1186/s13059-020-02066-4)
Supplement: Supplementary file 1 — Additional file 1 Contains supplementary text, figures, and tables. [file 13059_2020_2066_MOESM1_ESM.pdf]

A. Notation

For a graph G we write $V(G)$ and $E(G)$ for its vertex and edge set, respectively. For a vertex $v \in V(G)$, we write $N(v)$ to denote its neighborhood, i.e. the set containing all vertices that are adjacent to it in G. We further write $G - v$ for the graph obtained from G by removing v and all edges incident to it.

For a directed graph (digraph) $\vec{G}$, we use $N^-(v)$ to denote the *in-neighborhood* of $v \in V(\vec{G})$, that is, the set of all vertices w for which an arc wv exists in $\vec{G}$. The size of that in-neighbourhood is called the *in-degree* of v. We write $\Delta^-(\vec{G})$ for the maximum in-degree over all vertices of $\vec{G}$. For ease of notation, we usually write "$v \in G$" instead of "$v \in V(G)$" and "$uv \in G$" instead of "$\{u, v\} \in E(G)$" (same for digraphs).

A vertex set $S \subseteq V(G)$ is *r-scattered* if all vertices in it have pairwise distance at least r in G. Note that if a set S is $(2r+1)$-scattered in G, then no single vertex can r-dominate more than one vertex of S. It follows that any r-dominating set of G must have size at least $|S|$; we can therefore use $(2r+1)$-scattered sets to prove lower bounds on the r-domination number of a graph.

B. Dtf-augmentation

For the sake of completeness, we briefly describe how dtf-augmentations $\vec{G}_1, \vec{G}_2, \dots, \vec{G}_r$ are computed for an input graph G.

First, the graph $\vec{G}_1$ is a *minimum in-degree orientation* of G obtained as follows: we find a vertex u of minimum degree in G, orient the incident edges towards u and the repeat the procedure in $G - u$ until no vertices remain.

Given $\vec{G}_i$, $i \geq 1$ we then compute $\vec{G}_{i+1}$ by applying an *augmentation step*:

1. $\vec{G}_{i+1}$ contains all arcs of $\vec{G}_i$,
2. if there is a pair of arcs $uv \in \vec{G}_j$ and $vw \in \vec{G}_k$ with $j+k = i+1$, then we add uw to $\vec{G}_{i+1}$,
3. if there is a pair of arcs $uv \in \vec{G}_j$ and $wv \in \vec{G}_k$ with $j+k = i+1$, then either the arc uw or the arc wu must be in $\vec{G}_{i+1}$.

The ambiguity in the last step is resolved as follows: we collect all edges uw for which the last case applies in a graph H and then compute a minimum in-degree orientation $\vec{H}$ of H. We then add the arcs in $\vec{H}$ to $\vec{G}_{i+1}$.

For ease of notation, we define $\omega(uv)$ to be the smallest index $i \leq r$ such that $uv \in \vec{G}_i$ (and ∞ if no such index exists). We make use of two crucial properties of dtf augmentations in the following. First, vertices that have distance at most r in G have distance at most two in $\vec{G}_r$:

Lemma 1 (c.f. Lemma 26 in (24))**.** *For every pair $u, v \in G$ with $dist(u, v) = d$ and for every integer $r \geq d$ one of the following holds:*

1. *$uv \in \vec{G}_r$ and $\omega(uv) = d$,*
2. *$vu \in \vec{G}_r$ and $\omega(vu) = d$,*
3. *or there exists x such that $xu, xv \in \vec{G}_r$ and $\omega(xu) + \omega(xv) = d$.*

Second, if we compute dtf-augmentations of graphs from a bounded expansion class (to which graphs of bounded maximum degree like cDBGs belong), then the maximum in-degree of its rth augmentation can be bounded by a function independent of the *size* of the graph:

Theorem 1 (c.f. Theorem 16 in (24))**.** *For every graph class $\mathcal{G}$ with bounded expansion there exists a function f such that for every member $G \in \mathcal{G}$ the rth dtf-augmentation $\vec{G}_r$ computed as above satisfies $\Delta^-(\vec{G}_r) \leq f(r)$.*

C. Approximation guarantee

Let us introduce some notation for the analysis of Algorithm 1. We first partition the vertices of D according to whether they were added in line 10 (denoted by D_1) or in line 15 (denoted by D_2). Let $v_1, \dots, v_n$ be the vertex- order in which they are iterated over in the loop starting at line 6. We will use the notation D_1^i, D_2^i, d^i, and c^i to represent the states of the respective sets and data structures during the ith iteration of said loop. Let $\tau :=$ `domThreshold`(r) be the chosen threshold (we discuss a good value for τ on cDBGs below).

Lemma 2. *After the for-loop at line 7 has finished,*

$$d^i[v_i] = \begin{cases} dist_G(v, D^i) & \text{if } dist_G(v, D^i) \leq r, \text{ and} \\ \infty & \text{otherwise.} \end{cases}$$

Proof. The statement trivially holds while $D^i = \emptyset$, so assume otherwise. Let $u_h \in D^i$ be the vertex closest to v_i and let $h < i$ be the iteration in which u_h was added to D (either in line 10 or line 15 of that iteration).

If $d := \text{dist}_G(v_i, u_h) > r$, then $d^i[v_i]$ has not been changed yet and is still set to ∞. Otherwise, consider the three possible scenarios promised by the distance-property of dtf-augmentations:

Case 1: $v_i u_h \in \vec{G}_d$. Then $\omega(v_i u_h) = d$ and in iteration h the value of $d^h[v_i]$ is set to the correct value d at line 8. By assumption this distance remains minimal until iteration i and hence $d^i[v_i] = d^h[v_i] = d$.

Case 2: $u_h v_i \in \vec{G}_d$. Then $\omega(v_i u_h) = d$ and in iteration i the value of $d^i[v_i]$ is set to the correct value d at line 8.

Case 3: $xu_h, xv_i \in \vec{G}_d$ with $\omega(xu_h) + \omega(xv_i) = d$. During iteration h the value of $d^h[x]$ is set to $\omega(xu_h)$ at line 8 and subsequently retrieved in iteration i when $d^i[v_i]$ is set to

$$d^i[x] + \omega(xu_h) = \omega(xu_h) + \omega(xv_i) = d.$$

We conclude that after the execution of the loop at line 8. $d^i[v_i]$ is set to ∞ if v_i is not dominated by D_i and is otherwise set to $\text{dist}_G(v_i, D^i)$, as claimed. □

As an immediate consequence, we see the conditional statement at the end of the loop at line 8 accurately determines whether v_i is dominated by D_i or not. Accordingly, line 15 of the loop is only executed if v_i is *not* dominated by D^i. Another consequence is that all vertices in D_1 have large distance to each other:

Corollary 1. *The set D_1 is $(r+1)$-scattered in G.*

We need one more important property of the algorithm in order to derive the approximation factor.

Lemma 3. *For every $w \in G$ it holds that $|D_1 \cap N_r^-(w)| \leq \tau + 1$.*

Proof. Assume towards a contradiction that $\tau + 2$ such vertices $v_{i_1}, \dots, v_{i_\tau+2}$, $i_1 < i_2 < \dots < i_{\tau+2}$ exist in $D_1 \cap N_r^-(w)$. Since every such vertex v_i, $i \in \{i_1, \dots i_{\tau+2}\}$, was added to D in part (2), part (3) of the algorithm was executed during iteration i as well. Thus $c[w]$ was increased in each iteration i and during iteration $i_{\tau+1}$ we have that $c[w] \geq \tau + 1$ after the increment of $c[w]$. Therefore part (4) must have been executed for w, including w into D. Hence $w \in D^s$ for $s > i_{\tau+1}$ and in particular $w \in D^{i_{\tau+2}}$. But then $v_{i_{\tau+2}}$ was dominated by w at the beginning of iteration $i_{\tau+2}$ since we assumed that $\omega(rv_{i_{\tau+2}}) \leq r$, thus $v_{i_{\tau+2}}$ would not have been included in D at step (2). This contradicts our assumption of $v_{i_{\tau+2}} \in D_1$ so the claim must hold. □

Lemma 4. *There exists a subset $A \subseteq D_1$ such that A is $(2r+1)$-scattered in G and*

$$|A| \geq \frac{|D|}{2(\tau+2)\Delta^-(\vec{G}_{2r}))\Delta^-(\vec{G}_r)}.$$

Proof. We construct an auxiliary graph H with vertices D_1 by adding arcs $v_i v_j$ for $v_i, v_j \in D_1$ with $i < j$ whenever $\text{dist}_G(v_i, v_j) \leq 2r$. Let $\vec{G}_{2r}$ be a $2r$th dtf-augmentation of G and let us create a digraph $\vec{H}$ by orienting every edge $uv \in H$ as follows:

1. If of $uv, vu \in \vec{G}_{2r}$, then orient uv in $\vec{H}$ according to the corresponding arc in $\vec{G}_{2r}$ (if both arcs exists choose an arbitrary orientation),

2. otherwise there exists $w \in N^-_{2r}(u) \cap N^-_{2r}(v)$ with $\omega_{2r}(u) + \omega_{2r}(v) = \text{dist}_G(u,v) \le 2r$. Orient the edge uv towards that vertex $x \in \{u,v\}$ for which $\omega_{2r}(x)$ is larger.

We now argue that $\Delta^-(\vec{H})$ is small. Consider any vertex $v \in \vec{H}$. Every in-arc $uv \in \vec{H}$ either is of type 1, of which we have at most $\Delta^-(\vec{G}_{2r})$, or of type 2. Consider a group of in- arcs $u_i v$, $1 \le i \le \ell$ of type 2 that are all present because of a common vertex w. Since $w \in N^-_{2r}(u)$, we have at most $\Delta^-(\vec{G}_{2r})$ such groups. By construction, $\omega_{2r}(wu_i) \le \omega_{2r}(wv)$ and since both weights sum to less than $2r$, this means that $\omega_{2r}(wu_i) \le r$. Lemma 3 now tells us that $\ell \le \tau + 1$. Therefore v has at most $(\tau+1)\Delta^-(\vec{G}_{2r})$ in-arcs of type 2, and we conclude that

$$\Delta^-(\vec{H}) \le \Delta^-(\vec{G}_{2r}) + (\tau+1)\Delta^-(\vec{G}_{2r}) = (\tau+2)\Delta^-(\vec{G}_{2r}).$$

This finally implies that H is $2(\tau+2)\Delta^-(\vec{G}_{2r})$-degenerate and therefore contains an independent set $A \subseteq V(H)$ of size at least $|A| \ge |H|/(2(\tau+2)\Delta^-(\vec{G}_{2r}))$. Taken together with the fact that $|H| = |D_1| \ge |D|/\Delta^-(\vec{G}_r)$ (every vertex added to D_1 will cause at most $\Delta^-(\vec{G}_r)$ many vertices to be added to D_2 in the loop at line 11 and $D = D_1 \cup D_2$), we find that

$$|A| \ge \frac{|D|}{2(\tau+2)\Delta^-(\vec{G}_{2r}))\Delta^-(\vec{G}_r)}$$

By construction of H we conclude that A is $(2r+1)$-scattered in G of the claimed size. □

Since a $(2r+1)$-scattered set provides a lower bound for an r-dominating set, we conclude that Algorithm 1 computes a $2(\tau+2)\Delta^-(\vec{G}_{2r})\Delta^-(\vec{G}_r)$-approximation of an optimal r-dominating set. In other words, we obtain a constant-factor approximation in graphs of bounded expansion since the quantities $\Delta^-(\vec{G}_{2r})\Delta^-(\vec{G}_r)$ are constants per Theorem 1 in bounded expansion classes and the quantity $2(\tau+2)$ is a constant of our choosing.

In practice one could, depending on the value of $\Delta^-(\vec{G}_r)$ and $\Delta^-(\vec{G}_{2r})$, compute the optimal value for τ to minimize the approximation guarantee. However, this would necessitate the computation of $2r$ augmentation, the expensive step we want to avoid. Alternatively, we can choose a 'good enough' value for τ that still guarantees a constant-factor approximation while being easy to determine in practice. In the context of cDBGs, we found that $\tau := (2r)^2$ yields reliably good results.

D. Computational Runtimes

See "Benchmarking" in Materials and Methods for benchmarking methods.

The **podarV** data set was retrieved from the NCBI SRA using accession SRR606249. The full build and indexing of the 103 million error-trimmed reads (10.3 Gbp in total) took approximately 23 minutes and required 12.8 GB of RAM. Loading the indices for search required 4.3 GB of RAM and a search with a 3 Mbp genome took approximately 32 seconds.

The **HuSB1** data set was retrieved from the NCBI SRA using accession SRR1976948. The full build and indexing of the 34 million error-trimmed reads (8.5 Gbp in total) required approximately 217 minutes and required 24.4 GB of RAM. Loading the indices for search required 18 GB of RAM and a search with a 3 Mbp genome took approximately 80 seconds.

For data set complexity (number of k-mers, number of cDBG nodes) please see Table 1.

E. spacegraphcats pipeline overview

spacegraphcats follows a series of steps when run on sequencing data, see Figure S1. In detail, we perform the following steps.

BCALM. Use BCALM to generate a cDBG. Then convert a BCALM `unitigs.fa` output (a cDBG) into spacegraphcats files. Outputs an undirected graph, a file containing the sequences, and a `.info.csv` file containing information about the contig. Also outputs sourmash `k=31`, `scaled=1000` signatures for both input and output files.

spacegraphcats.cdbg.label_cdbg. Build an index that can be used to retrieve individual reads or contigs by cDBG node ID; produce a SQLite database for fast retrieval. Briefly, this script creates a sqlite database with a single table, `sequences`, where a query like `SELECT DISTINCT sequences.offset FROM sequences WHERE label ...` can be executed to return the offset of all sequences with the given label; the offsets refer to BGZF coordinates in the gzipped sequence collection. Here, 'label' is the cDBG ID to which the sequence belongs.

The script `extract_reads_by_frontier_sqlite.py` is a downstream script to extract the reads with a frontier search. Specifically: 1. walk through the contigs assembled from the cDBG; 2. build a DBG cover using khmer tags, such that every k-mer in the DBG is within distance d=40 of a tag; 3. label each tag with the cDBG node ID from the contig; 4. save for later use.

spacegraphcats.catlas.catlas. The catlas is a hierarchical atlas for querying graphs. Implements algorithms 1 and 2 (see main text).

spacegraphcats.index.index_contigs_by_kmer. Use Minimal Perfect Hashing (BBHash, https://github.com/rizkg/BBHash) to construct a fast lookup table connecting k-mers in the cDBG to cDBG node IDs. (BBHash reference: A. Limasset, G. Rizk, R. Chikhi, P. Peterlongo, Fast and Scalable Minimal Perfect Hashing for Massive Key Sets, SEA 2017.)

spacegraphcats.search.extract_nodes_by_query. Do a frontier search, and retrieve cDBG node IDs and MinHash signature for the retrieved contigs.

spacegraphcats.search.extract_contigs. Retrieve the unitig sequences for a given list of cDBG nodes. Consumes the output of `extract_nodes_by_query` to get the list of nodes.

spacegraphcats.search.extract_reads. Retrieve the reads for a list of cDBG nodes. Consumes the output of `extract_nodes_by_query` to get the list of nodes, and then uses the labeled cDBG output by `.cdbg.label_cdbg` to find reads that overlap with the unitigs in those nodes.

F. Query genome accession numbers for *Proteiniclasticum* search

See Table S1.

G. Amino Acid Identity results for *Proteiniclasticum*

See Table S2.

H. HuSB1 analysis pipeline overview

See Figure S2. We implemented three workflows to analyze the plass-assembled **HuSB1** query neighborhoods.

Name	NCBI accession
P. ruminis CGMCC	GCA_900099635.1
P. ruminis DSM	GCA_000701905.1
P. ruminis ML2	GCA_900115135.1

Table S1. Accession numbers for genomes used in *Proteiniclasticum* neighborhood query.

Genome A	Genome B	Orthologous Genes	Mean AAI
P. ruminis ML2	*P. ruminis shakya*	2546	95.74
P. ruminis DSM	*P. ruminis shakya*	2391	93.47

Table S2. CompareM results for *Proteiniclasticum* genomes. *P. ruminis shakya* is the result of assembling the reads extracted from podarV with the neighborhood search.

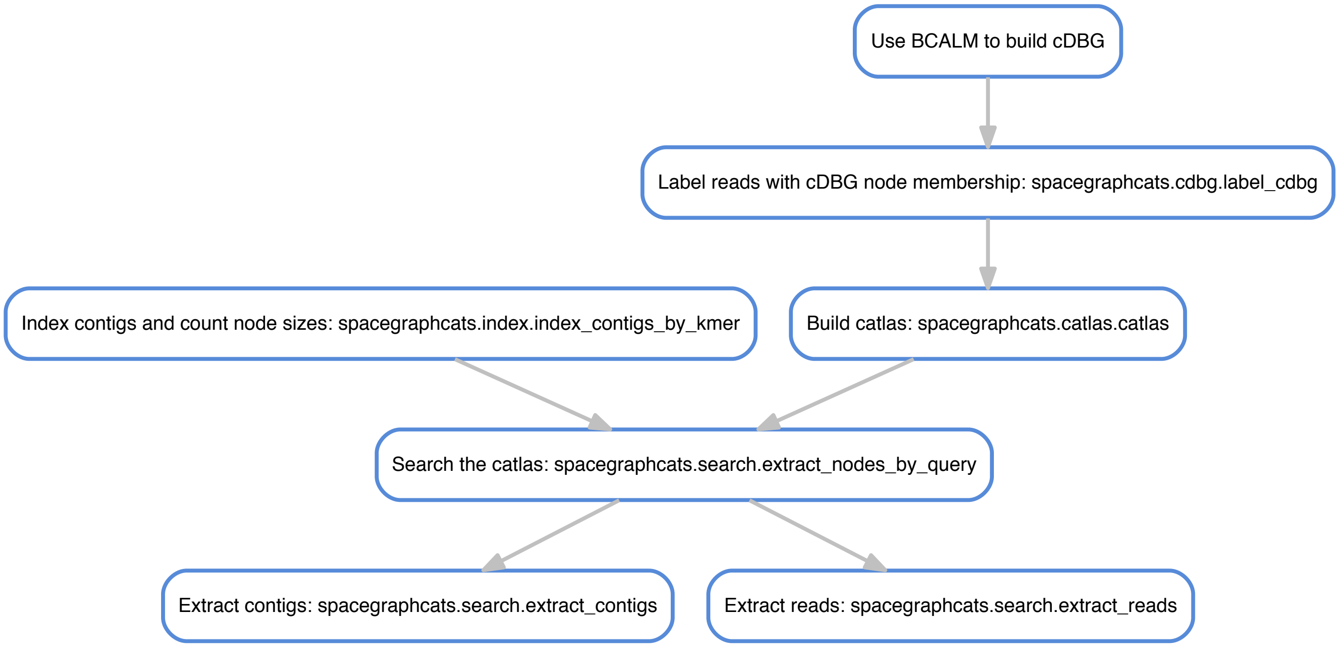

Fig. S1. The steps followed by spacegraphcats when run on sequencing data.

I. Genome bin completeness improvements for `HuSB1`

See Table S3 and Table S4. Table S3 shows completeness metrics for the binned genomes from the `HuSB1` sample and used as queries. Table S4 shows completeness metrics for Plass assembled query neighborhoods after stringent read trimming at low abundance k-mers (k-mers present fewer than 5 times were removed) of the SB1 sample reads.

J. K-mer inclusion of reads by MEGAHIT assemblies

See Table S6. We estimated the number of k-mers in each query neighborhood that were contained in the MEGAHIT assembly of that query neighborhood. We used sourmash `compute` to calculate signatures with k-size of 31 and a scaled value of 2000. We then used sourmash `compare` to estimate containment in MEGAHIT assemblies. The query neighborhood with the smallest containment, *M. harundinacea* isolate *57_489*, had the largest query neighborhood, while the query neighborhood with the largest containment, *M. bacterium 39_7*, had the smallest query neighborhood.

K. gyrA alignment

See Figure S3. The MDS plot in the left panel of figure 4 shows distinct gyrA sequences identified in the Plass assemblies using HMMER. To visualize the sequences within these clusters and in other query neighborhoods, we constructed a multiple sequence alignment. However, because many sequences assembled by Plass were fragmented (see Results: Some query neighborhoods contain substantial strain variation), we first clustered the sequences at 95% similarity using CD-HIT. We then aligned the centroid sequences using MAFFT with default settings. To produce the multiple sequence alignment visualization, we calculated an unrooted neighbor joining tree using the MAFFT alignment. Then we used the function `msaplot` in the R package ggtree to plot the alignment.

L. gyrA by neighborhood

See Table S5. As can be seen in the left panel of Figure 4 in the main text, we observe many unique amino acid sequences per single copy ortholog per query neighborhood. Although we observe many possible traversal paths in compact De Bruijn graphs built from reads that give rise to these sequences, we have no way to ascertain whether we observed combinatorial complexity by assembling variants that would never be linked in nature. Therefore, we sought to conservatively estimate the number of positions per amino acid sequence that contained variants using MAFFT alignments. First, we subsetted the alignment to sequences from one query neighborhood. Then we identified all aligned non-gap characters for each position in the alignment (gaps were induced in some neighborhoods by the presence of amino acid residues in other query neighborhood amino acid sequences). For each of these positions, we counted the number of unique amino acid sequences per position, and the number of times each occurred at that position. We then elimated any variant that occurred fewer than 10 times. Lastly, we counted the number of well-supported distinct characters. We did this for gyrA, as well as the amino acid sequences for the other genes we tested (see other genes). Table S5 shows that we see increased number of gyrA sequences in many neighborhoods even with this conservative approach.

M. Other genes

See bin and neighborhood content results for *alaS* in Table S8, *gyrB* in Table S9, *recA* in Table S10, *rpb2d6* in Table S11, rplB in Table S12, and rpsC in Table S13. We selected *gyrB* and *recA* because they were used by `HuSB1` to assign taxonomy to binned genomes. We selected other genes used as single copy orthologs by programs like CheckM, and with longer PFAM domains.

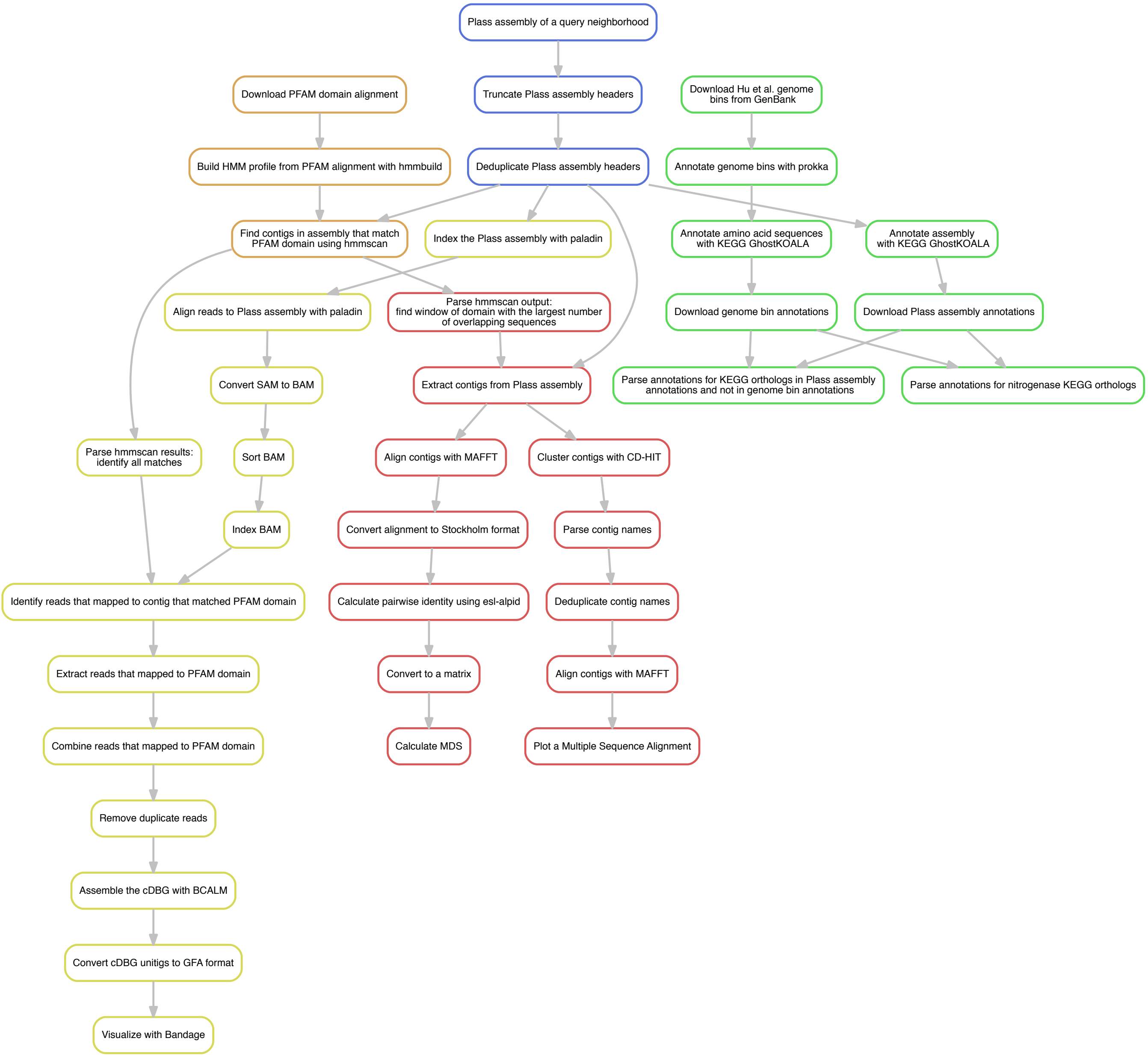

Fig. S2. Three workflows implemented to analyze the plass-assembled `HuSB1` query neighborhoods. The first three steps, depicted in blue, were common across all workflows. The green boxes depict the KEGG GhostKOALA annotation workflow, the results of which can be see in Figure 4. The orange boxes depict steps in common between the clustering and variant workflows used to generate Figure 4. The red boxes depict steps used to generate the MDS clustering plot and the multiple sequence alignment (see Figure S3). The gold boxes depict the steps of the variant workflow used to generate the assembly graphs.

Species	Completeness (%)	Redundancy (%)	Strain heterogeneity (%)	Unique KOs	Size (bases)	Number of proteins
WS6 bacterium 36_33	31.5	6.5	85.7	127	439774	423
P. bacterium 34_609	34.0	1.1	12.5	173	567617	557
P. bacterium 33_209	47.9	5.2	0	140	510490	526
M. bacterium 39_7	50.1	5.9	0	208	708389	657
P. acetatigenes isolate 50_10	56.7	3.6	66.7	506	1716233	1510
WS6 bacterium 34_10	61.9	29.5	61.3	219	1127556	1065
M. infera isolate 46_47	63.8	0.9	100	502	1225111	1170
A. bacterium 34_128	64.4	5.1	0	423	894916	785
A. thermophila isolate 46_16	67.2	16.4	0	474	1524726	1350
A. bacterium 49_20	69.6	8.3	0	380	1023183	891
M. marisnigri isolate 62_101	72.1	1.0	0	583	1592820	1742
M. bacterium 46_47	72.9	0.1	0	563	1629409	1413
B. bacterium 38_7	80.0	2.7	0	601	2137321	1697
Methanocalculus sp. 52_23	82.7	4.6	66.7	689	1973787	2074
Desulfotomaculum sp. 46_80	83.5	2.5	42.9	834	2251381	2148
S. bacterium 57_84	90.8	1.9	50	608	1255134	1277
S. bacterium 53_16	91.5	4.2	0	746	1772227	1741
Desulfotomaculum sp. 46_296	91.5	9.0	65.4	851	2328136	2265
A. bacterium 66_15	94.2	2.8	20	811	2228088	2185
C. bacterium 38_11	94.4	0.3	0	825	1882878	1744
TA06 bacterium 32_111	94.5	0	0	619	1861827	1736
Methanobacterium sp. 42_16	97.6	0	0	769	2173293	2149
M. harundinacea isolate 57_489	100	0	0	814	2382964	2377

Table S3. Completeness metrics for the `HuSB1` genome bins from (35), used as queries into the SB1 metagenome. Completeness, redundancy, and strain heterogeneity as estimated by checkM, unique KEGG orthologs predicted by GhostKOALA, bin size in bases, and number of prokka-predicted protein sequences in the `HuSB1` bins. Table is ordered by completeness. Note we refer to the checkM term "contamination" as "redundancy" as this better describes the calculated metric.

Species	Completeness (%)	Redundancy (%)	Strain heterogeneity (%)	Unique KOs	Size (bases)	Number of proteins
WS6 bacterium 36_33	45.7	1675.8	87.9	140	1206915	12990
P. bacterium 34_609	39.7	637.3	74.8	239	3604616	12372
P. bacterium 33_209	46.0	516.1	92.7	160	1830403	9836
M. bacterium 39_7	30.8	1.9	20	121	1356859	1208
P. acetatigenes isolate 50_10	42.7	66.3	86.7	557	6829683	6135
WS6 bacterium 34_10	58.6	352.8	86.0	219	2840498	6549
M. infera isolate 46_47	67.0	309.8	92.6	592	4021633	11798
A. bacterium 34_128	65.5	332	58.9	446	2703261	9030
A. thermophila isolate 46_16	60.5	250	88.5	457	5080416	13780
A. bacterium 49_20	71.3	187.2	76.7	392	4166615	6040
M. marisnigri isolate 62_101	82.2	1067.9	89.9	687	11380474	49119
M. bacterium 46_47	81.0	251.3	97.5	629	5308863	15964
B. bacterium 38_7	44.0	5.3	20	508	4229976	3901
Methanocalculus sp. 52_23	89.3	443.4	87.0	741	8018167	20999
Desulfotomaculum sp. 46_80	94.8	2327.3	94.7	858	7969109	48841
S. bacterium 57_84	90.8	598.2	83.9	686	5968936	24774
S. bacterium 53_16	94.0	95.6	83.9	786	7145072	12138
Desulfotomaculum sp. 46_296	94.8	2535.0	80.4	888	17836454	48174
A. bacterium 66_15	83.6	10.6	83.7	792	10865439	8152
C. bacterium 38_11	86.7	18.2	78.4	800	4932373	4582
TA06 bacterium 32_111	95.6	45.7	88.2	626	3811703	5724
Methanobacterium sp. 42_16	92.2	31.3	90.9	782	7007709	6335
M. harundinacea isolate 57_489	98.8	355.6	89.6	841	35722445	35093

Table S4. Completeness metrics for the query neighborhoods extracted from the `HuSB1` metagenome by spacegraphcats**. Completeness, redundancy, and strain heterogeneity as estimated by checkM, unique KEGG orthologs predicted by GhostKOALA, neighborhood size in bases, and number of plass-assembled protein sequences in the query neighborhoods. Table is ordered by completeness of query bins (see Table S3). All estimates were performed on the k-mer trimmed (k >= 5) Plass-assembled proteins except size of neighborhood in bases, for which we used the neighborhood unitig sequences output by** spacegraphcats**.**

Species	gyrA (bin)	gyrA (Plass)
Methanobacterium sp. 42_16	0	0
P. bacterium 34_609	0	0
Desulfotomaculum sp. 46_80	0	0
S. bacterium 57_84	0	0
B. bacterium	0	1
P. acetatigenes isolate 50_10	0	2
WS6 bacterium 34_10	0	2
M. marisnigri isolate 62_101	0	2
C. bacterium 38_11	1	1
M. infera isolate 46_47	1	1
S. bacterium 53_16	1	1
M. bacterium 46_47	1	1
TA06 bacterium 32_111	1	1
P. bacterium 33_209	1	1
A. bacterium 66_15	1	1
Methanocalculus sp. 52_23	1	2
WS6 bacterium 36_33	1	2
A. bacterium 34_128	1	2
A. thermophila isolate 46_16	1	2
M. harundinacea isolate 57_489	1	2
M. bacterium 39_7	2	0
Desulfotomaculum sp. 46_296	2	2
A. bacterium 49_20	2	3

Table S5. Bin and neighborhood gyrA protein content. gyrA count for each bin is the number of gyrA amino acid sequences that are part of the original bin. gyrA count by Plass is the minimum number of gyrA amino acid sequences supported by at least one position with at least 10 copies of each variant, e.g., "3" indicates that there is at least one position in the multiple sequence alignment of gyrA sequences for that neighborhood that has 3 distinct variants in 10 distinct sequences.

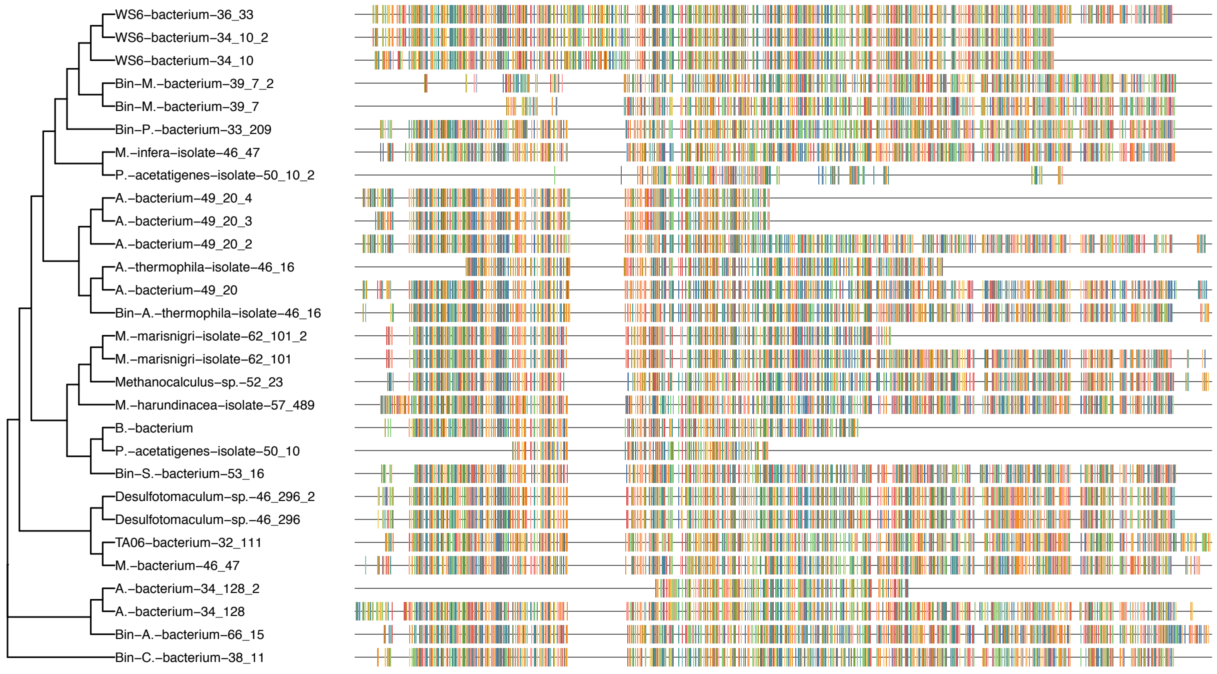

Fig. S3. A multiple sequence alignment and neighbor joining tree of representative gyrA amino acid fragments assembled by Plass from the genome neighborhoods in `HuSB1`. Protein sequences that originated from the genome bin are prepended with "Bin." All other sequences were assembled by Plass.

Species	MEGAHIT assembly containment
M. harundinacea isolate 57_489	4.2%
Desulfotomaculum sp. 46_296	12.7%
M. marisnigri isolate 62_101	13.6%
S. bacterium 57_84	19.4%
P. bacterium 34_609	19.7%
A. bacterium 66_15	20.5%
Desulfotomaculum sp. 46_80	24.1%
P. bacterium 33_209	26.3%
S. bacterium 53_16	30.9%
A. bacterium 49_20	31.9%
Methanocalculus sp. 52_23	33.4%
M. bacterium 46_47	36.6%
P. acetatigenes isolate 50_10	36.6%
A. bacterium 34_128	36.8%
M. infera isolate 46_47	38.0%
Methanobacterium sp. 42_16	38.0%
A. thermophila isolate 46_16	38.6%
TA06 bacterium 32_111	44.1%
C. bacterium 38_11	44.4%
WS6 bacterium 34_10	53.2%
WS6 bacterium 36_33	53.8%
B. bacterium	54.2%
M. bacterium 39_7	55.7%

Table S6. Containment of neighborhood k-mer content in MEGAHIT nucleotide assemblies.

Name	PFAM accession
recA	PF00154
rplB	PF00181
rpsC	PF00189
gyrB	PF00204
gyrA	PF00521
rpb2d6	PF00562
alaS	PF01411

Table S7. Protein names and PFAM accessions for targeted analyses.

Species	alaS (bin)	alaS (Plass)
P. acetatigenes isolate 50_10	0	0
A. bacterium 49_20	0	0
P. bacterium 34_609	0	0
B. bacterium	0	0
S. bacterium 53_16	0	0
A. bacterium 34_128	0	0
M. infera isolate 46_47	0	2
M. marisnigri isolate 62_101	0	2
M. bacterium 39_7	1	0
Methanobacterium sp. 42_16	1	1
C. bacterium 38_11	1	1
S. bacterium 57_84	1	1
TA06 bacterium 32_111	1	1
P. bacterium 33_209	1	1
A. bacterium 66_15	1	1
M. harundinacea isolate 57_489	1	1
Methanocalculus sp. 52_23	1	2
WS6 bacterium 36_33	1	2
Desulfotomaculum sp. 46_80	1	2
M. bacterium 46_47	1	2
Desulfotomaculum sp. 46_296	1	2
A. thermophila isolate 46_16	2	1
WS6 bacterium 34_10	2	2

Table S8. Bin and neighborhood alaS protein content.

Species	gyrB (bin)	gyrB (Plass)
M. bacterium 39_7	0	0
P. acetatigenes isolate 50_10	0	0
Methanobacterium sp. 42_16	0	0
WS6 bacterium 36_33	0	0
P. bacterium 34_609	0	0
Desulfotomaculum sp. 46_80	0	0
S. bacterium 57_84	0	0
S. bacterium 53_16	0	0
A. thermophila isolate 46_16	0	2
P. bacterium 33_209	1	0
C. bacterium 38_11	1	1
M. infera isolate 46_47	1	1
M. bacterium 46_47	1	1
TA06 bacterium 32_111	1	1
A. bacterium 66_15	1	1
M. harundinacea isolate 57_489	1	1
WS6 bacterium 34_10	1	2
Methanocalculus sp. 52_23	1	2
M. marisnigri isolate 62_101	1	2
A. bacterium 34_128	1	2
A. bacterium 49_20	2	2
B. bacterium	2	2
Desulfotomaculum sp. 46_296	2	2

Table S9. Bin and neighborhood gyrB protein content.

Species	recA (bin)	recA (Plass)
M. bacterium 39_7	0	0
WS6 bacterium 34_10	0	0
Methanocalculus sp. 52_23	0	0
A. bacterium 49_20	0	0
WS6 bacterium 36_33	0	0
P. bacterium 34_609	0	0
M. marisnigri isolate 62_101	0	0
S. bacterium 53_16	0	0
M. bacterium 46_47	0	0
A. thermophila isolate 46_16	0	1
B. bacterium	1	0
P. acetatigenes isolate 50_10	1	1
Methanobacterium sp. 42_16	1	1
C. bacterium 38_11	1	1
M. infera isolate 46_47	1	1
S. bacterium 57_84	1	1
A. bacterium 34_128	1	1
TA06 bacterium 32_111	1	1
P. bacterium 33_209	1	1
A. bacterium 66_15	1	1
M. harundinacea isolate 57_489	1	1
Desulfotomaculum sp. 46_80	1	2
Desulfotomaculum sp. 46_296	1	2

Table S10. Bin and neighborhood recA protein content.

Species	rpb2d6 (bin)	rpb2d6 (Plass)
P. acetatigenes isolate 50_10	0	0
P. bacterium 34_609	0	0
S. bacterium 57_84	0	1
M. bacterium 46_47	0	1
C. bacterium 38_11	1	0
A. bacterium 49_20	1	0
M. bacterium 39_7	1	1
Methanobacterium sp. 42_16	1	1
Methanocalculus sp. 52_23	1	1
M. infera isolate 46_47	1	1
B. bacterium	1	1
S. bacterium 53_16	1	1
A. bacterium 34_128	1	1
TA06 bacterium 32_111	1	1
A. bacterium 66_15	1	1
A. thermophila isolate 46_16	1	1
WS6 bacterium 36_33	1	2
Desulfotomaculum sp. 46_80	1	2
M. marisnigri isolate 62_101	1	2
Desulfotomaculum sp. 46_296	1	2
P. bacterium 33_209	1	2
M. harundinacea isolate 57_489	1	2
WS6 bacterium 34_10	2	2

Table S11. Bin and neighborhood rpb2d6 protein content.

Species	rplB (bin)	rplB (Plass)
M. bacterium 39_7	0	0
Methanobacterium sp. 42_16	0	0
Methanocalculus sp. 52_23	0	0
WS6 bacterium 36_33	0	0
M. marisnigri isolate 62_101	0	0
M. harundinacea isolate 57_489	0	0
P. acetatigenes isolate 50_10	1	1
C. bacterium 38_11	1	1
A. bacterium 49_20	1	1
M. infera isolate 46_47	1	1
P. bacterium 34_609	1	1
Desulfotomaculum sp. 46_80	1	1
S. bacterium 57_84	1	1
B. bacterium	1	1
S. bacterium 53_16	1	1
A. bacterium 34_128	1	1
M. bacterium 46_47	1	1
Desulfotomaculum sp. 46_296	1	1
TA06 bacterium 32_111	1	1
P. bacterium 33_209	1	1
A. bacterium 66_15	1	1
A. thermophila isolate 46_16	1	1
WS6 bacterium 34_10	1	2

Table S12. Bin and neighborhood rplB protein content.

Species	rpsC (bin)	rpsC (Plass)
M. bacterium 39_7	0	0
P. acetatigenes isolate 50_10	0	0
WS6 bacterium 34_10	0	0
Methanobacterium sp. 42_16	0	0
Methanocalculus sp. 52_23	0	0
WS6 bacterium 36_33	0	0
M. marisnigri isolate 62_101	0	0
B. bacterium	0	0
P. bacterium 33_209	0	0
M. harundinacea isolate 57_489	0	0
M. infera isolate 46_47	0	1
C. bacterium 38_11	1	1
A. bacterium 49_20	1	1
P. bacterium 34_609	1	1
S. bacterium 57_84	1	1
S. bacterium 53_16	1	1
A. bacterium 34_128	1	1
M. bacterium 46_47	1	1
TA06 bacterium 32_111	1	1
A. bacterium 66_15	1	1
A. thermophila isolate 46_16	1	1
Desulfotomaculum sp. 46_80	1	2
Desulfotomaculum sp. 46_296	1	2

Table S13. Bin and neighborhood rpsC protein content.
