# Supplementary material for: Exploring neighborhoods in large metagenome assembly graphs using spacegraphcats reveals hidden sequence diversity
Source: Genome Biol. 2020 Jul 6;21:164. doi: 10.1186/s13059-020-02066-4 (PMC7336657; doi:10.1186/s13059-020-02066-4)
Supplement: Supplementary file 2 — Additional file 2 Contains the review history. [file 13059_2020_2066_MOESM2_ESM.docx]

Review History

**First round of review**

**Reviewer 1**

**Were you able to assess all statistics in the manuscript, including the appropriateness of statistical tests used?** Yes. The paper does not describe any statistical analysis.

**Were you able to directly test the methods?** No.

**Comments to author:**

The authors present a fast method to explore the surrounding of a genome in a metagenome assembly graph. They apply their method to extend metagenomic bins and for the identification of strain variation. The paper is well written, and the new algorithm represent a significant time improvement in comparison to best known algorithm. I have some suggestion concerning the method performance evaluation.

Major comments:

For the assembly of the novel Proteiniclasticum, the author report an assembly with 100% completeness but with a contamination of 10% which is higher than the accepted threshold of 5% for MAG assemblies. It would be great to know the performance of a simple approach that: (1) identify Proteiniclasticum reads by mapping to the 3 known Proteiniclasticum genome, (2) assemble the reads.

In the analysis of the bin extension of the HuSB1 dataset, the authors only focused on the bin completeness. Other important statistics like bin size, number of genes assembled by PLASS and bin contamination should be reported.

The author mentioned that it is challenging to evaluate their method in a complex real metagenome in the absence of ground truth. A way to overcome this problem, will be to create a "virtual microbiome" (as described in "Nanopore sequencing enables high-resolution analysis of resistance determinants and mobile elements in the human gut microbiome") by pooling the sequencing reads from a complex real metagenome and an isolate/simple mock community for which the genome(s) are known. This will allow to create a complex dataset with known ground truth and allow to evaluate if the binning extension approach is able to rescue bins in the presence of multiple strains of the same species.

Minor comment:

Figure 3(b) the y-axis should be "completeness" instead of "complete".

**Reviewer 2**

**Were you able to assess all statistics in the manuscript, including the appropriateness of statistical tests used?** Yes. No further assessment of statistics is required (as it is not too applicable for this manuscript).

**Were you able to directly test the methods?** Yes.

**Comments to author:**

Summary:
This manuscript details an efficient method by which metagenomic assembly graphs can be queried and explored to extract sequences and subgraphs of interest. This is an important contribution given that most metagenomic (and for that matter, genomic) assembly graphs are viewed as an intermediate step and are frequently discarded in favor of the (often heuristically) generated contigs that are computed by the various assembly algorithms. Information is inevitably lost by reducing an assembly graph to a set of (linear) contigs, as the graph structure is discarded when contigs are generated. In contrast, this manuscript convincingly argues that considering neighborhoods of the assembly graph can significantly improve not just metagenomic binning efforts but can also provide additional biological insight. For example, analyzing certain extracted neighborhoods can shed light on novel organisms in a metagenome, improve functional annotation of metagenomes, and illustrate the strain variation present in real-world samples. I applaud the authors for their work in effectively communicating the basic idea of dominating sets in Figure 1, as this figure elegantly summarizes the basic idea of the paper.

The main technical contribution that facilitates the exploration of assembly graph neighborhoods is the concept of a "dominating set": sets of nodes in a (compact) de Bruijn graph that are guaranteed to be within short graph distance to all nodes of the graph. This allows an index to be created that enables efficient queries and neighborhood extraction. The authors provide a proof that their linear-time dominating set generation algorithm returns a dominating set of size within a constant factor to the (NP-hard to calculate) optimal dominating set size. In practice, it appears that this algorithm is quite efficient, and results in an index that allows for fast queries (see Table 2) with total execution time under an hour.

In all, I believe this computational package, called spacegraphcats, will serve to further illuminate the complex metagenomic communities that are observed in the environment. The ability to efficiently extract and explore particular neighborhoods of an assembly graph will also allow researchers to focus on particular portions of the metagenome of interest without relying on a global assembly method that attempts to heuristically assemble an entire metagenome. The authors mention further interesting potential applications of this approach in the conclusion, and justifiably position this approach as an additional source of information when compared to sequence composition, contig abundance, and marker gene approaches. I believe this is an important contribution to the metagenomic literature, and potentially applicable to other fields as well (where large graph queries are relevant).

Software review:
I was able to get the software package installed (using Ubuntu) and the provided small test to run without any problem. Note that the line ``pip install https://github.com/dib-lab/khmer/archive/master.zip`` in https://github.com/spacegraphcats/spacegraphcats/blob/master/doc/installing-spacegraphcats.md is unnecessary as the requirements file https://github.com/spacegraphcats/spacegraphcats/blob/master/requirements.txt already contains the line ``https://github.com/dib-lab/khmer/archive/master.zip``
I was also able to create my own workflow using an HMP mock community, and the software performed as expected (save for my initially overlooking the instruction to install BCALM 2; you may consider printing a message mentioning this given a standard user's penchant for copy-pasting install code from the *.md page).

There does remain, however, a few issues that could serve to improve the manuscript and facilitate its acceptance in the community.
Major comments:
I see three issues that may contribute to confusion to a reader of this manuscript, detailed below in descending order of importance.

First, while I understand that the target venue is not a mathematical/theoretical computer science journal, the details regarding the algorithms (and especially the proofs) assume quite a bit of background knowledge and make them somewhat difficult to parse. For example, the "distance-truncated transitive fraternal (dtf) augmentations" play a key role in Algorithm 1. However, the half-sentence description of what these are is insufficient to provide the reader with an understanding of the what a dtf augmentation actually does. The reference provided (24) is a 298 page thesis that provides little further understanding without significant investment of time on the part of the reader. Consider adding another sentence or two describing what these are, as the current description does not describe what the nodes and edges of the directed graph are that results from a dtf augmentation, nor makes clear whether $\omega(uv)$ is computed for all pairs of vertices $(u,v)$ or only for some. This also makes Algorithm 1 difficult to interpret as $\vec{G}_i \leftarrow \verb|dtfAugmentation|(\vec{G}_{i-1})$ leaves the reader wondering how the graph $\vec{G}_i$ has actually been transformed. Further issues in this regard include undefined notation: in Algorithm 2, the symbol $N(v)$ is used without any indication of what this is meant to represent. I image it's a neighborhood of sorts, but this should be made clear. This notation issue comes up again in the Appendix, where the claims and proofs are relatively straightforward to follow up until Lemma 3 where the mysterious symbol $\Delta^-(\cdot)$ is introduced. Not only is it not described what $\Delta^-$ is, but this is then applied to $\vec{G_{2r}} which is supposed to be a "2rth dtf-augmentation of $G$." Given none of these are defined or explained, it results in Lemma 3 being practically uninterpretable. Further uses of undefined terms include the repeated reference to graphs with "bounded expansion." From my understanding, bounded expansion is a property of a family of graphs, not a single graph. The manuscript does not make clear that compact de Bruijn graphs (as a family) have this property. Similarly, the claim near the end of Lemma 3 (page 10, line 32, column 2) makes the claim that their proof of a constant-factor approximation holds in graphs of bounded expansion without supporting this claim.

Second, it may serve the reader well to have the following sentence near the front of the paper instead of relegated to the second to last paragraph of the conclusion "In this initial investigation of neighborhood indexing, we have focused on using neighborhood queries with a genome bin." As I read through the paper, I began to wonder why the authors were focusing so heavily on genome bins when I could imagine other relevant queries. This was not clarified (that this was a choice for the initial investigation) until the end of the paper. A reader may be well-served to understand that one can query with more than just a genome bin earlier in the manuscript. Along those lines, to further communicate how genome binning can be improved with neighborhood queries (though I understand this is by far not the only application of their method), the authors may consider using their approach (assemble, bin, query with bins, use extracted neighborhoods to assemble new bins) along with recent binning benchmarking software (https://academic.oup.com/gigascience/article/7/6/giy069/5034950) to show improvements beyond their analysis of completeness and gyrA analysis. Personally, I would be interested in seeing if other binning metrics (such as purity) are improved with the author's approach.

Lastly, the section on page 5 titled "Query neighborhoods assembled with Plass contain additional functional content" is difficult to follow. For example, "subtract(ing) any homolog already annotated in the genome bin" leaves the reader wondering where the homologs were subtracted from (from the possibly Plass assembled neighborhood? From the KEGG database? From the genome bin?). A bit more description about what is being performed here would be helpful.
Minor comments:

Page 1, Line 35 "genic content" is, in my experience, a bit non-standard, so consider replacing with "genomic" or "genetic" content depending on the intent.

Figure 2: the colored lines in the left panel are not described. I assume they are linear regression lines, but it would be nice to have this explicitly stated.

Figure 2: in the right panel, it is mentioned that "recovery is calculated as containment of target genome in query neighborhood." Is this meant to indicate containment on the basis of k-mers? If so, is this calculated in a brute force fashion? With a particular k-mer size? With sourmash and k=31 as briefly mentioned in the supplementary material? Furthermore, containment is not the only relevant metric here, as the neighborhoods extracted could be quite large (i.e. |neighborhood| - |target genome| is large even though |target genome \intersect neighborhood| / | target genome| is small). Perhaps mention the relative size of the neighborhood in comparison to the target genome (and describe if this is done at a particular k-mer size, etc.).

Page 4, line 51-52: I found the sentence "However, we found little improvement in the completion metrics" confusing as figure 3(b) appears to show significant improvement in the completion metric. Indeed, elsewhere in the paper the data in figure 3(b) is used to support the claim that "Neighborhood queries extend genome bins." Please clarify the contradiction that you observe "little improvement in the completion metric" and yet (page 6, lines 19-32) you find an "increase in the estimated completeness of most genome bins."

Page 5, line 57 "ignoreed" typo

Page 5, line 48, column 2 "…useful to analyze sequence that has small graph distance…" consider "useful to analyze sequences that have small graph distance"

Page 7, lines 33-34, column 1 "…and then contracting long degree-two paths when possible." How does one contract a degree two path? Was a decision made to select one of the outgoing edges over another? This was unclear to me.

Appendix A, page 11, line 15, column 1. There is no reference provided for BBHash. Consider providing one

**Authors Response**

**Point-by-point responses to the reviewers’ comments:**

\# Response to Reviewers - spacegraphcats

**We thank the reviewers for their in depth and positive reviews! We have made many changes in response to the reviewers, and the full diff is available at** [**https://github.com/spacegraphcats/2018-paper-spacegraphcats/pull/14**](https://github.com/spacegraphcats/2018-paper-spacegraphcats/pull/14)**.**

Reviewer #1:

*The authors present a fast method to explore the surrounding of a genome in a metagenome assembly graph. They apply their method to extend metagenomic bins and for the identification of strain variation. The paper is well written, and the new algorithm represent a significant time improvement in comparison to best known algorithm. I have some suggestion concerning the method performance evaluation.*

**Thank you!**

*Major comments:

For the assembly of the novel Proteiniclasticum, the author report an assembly with 100% completeness but with a contamination of 10% which is higher than the accepted threshold of 5% for MAG assemblies. It would be great to know the performance of a simple approach that: (1) identify Proteiniclasticum reads by mapping to the 3 known Proteiniclasticum genome, (2) assemble the reads.*

**We thank the reviewers for this suggestion. When we assembled the reads that map to the available genomes, a marker-based analysis with CheckM estimates that 86.9% of the Proteiniclasticum variant is recovered (2.82% contamination, 0% strain heterogeneity). This improves upon the shared-k-mer approach we previously used.**

*In the analysis of the bin extension of the HuSB1 dataset, the authors only focused on the bin completeness. Other important statistics like bin size, number of genes assembled by PLASS and bin contamination should be reported.*

**We have included these results in the Appendix as Table 5 (analysis of original bins) and Table 6 (analysis of Plass-assembled neighborhoods). We chose not to further investigate the CheckM metrics of redundancy and contamination in this paper, as we wanted to first focus on whether the strain variation present in the expanded neighborhoods represented real or surprising biological content. (See also our discussion of containment vs similarity metrics in response to Reviewer 2.)**

**One future avenue of research with spacegraphcats could be to investigate and refine metrics and cutoffs for redundancy and contamination calculations. We note that these metrics are challenging to interpret and a thorough investigation would need to involve adjusting cutoffs for specific genes (see e.g. https://github.com/Ecogenomics/CheckM/issues/127#issuecomment-350893962 for some discussion of this).**

*The author mentioned that it is challenging to evaluate their method in a complex real metagenome in the absence of ground truth. A way to overcome this problem, will be to create a "virtual microbiome" (as described in "Nanopore sequencing enables high-resolution analysis of resistance determinants and mobile elements in the human gut microbiome") by pooling the sequencing reads from a complex real metagenome and an isolate/simple mock community for which the genome(s) are known. This will allow to create a complex dataset with known ground truth and allow to evaluate if the binning extension approach is able to rescue bins in the presence of multiple strains of the same species.*

**Thank you for this excellent suggestion! A similar evaluation strategy was used by us in Howe et al., 2014, https://www.pnas.org/content/111/13/4904.long, where we “spiked in” multiple known E. coli genomes to a soil metagenome to evaluate species recovery. We think this strategy would be an excellent way to evaluate the effect of different parameters (radius, k size) on genome recovery.**

**One significant design challenge for proper spacegraphcats benchmarking is that we would need to use a variety of strain and species query genomes in order to evaluate the changes in neighborhood structure at these different ANIs. However, it would be challenging to find real metagenomes that contain species with significant representation in cultured genome databases - we would probably have to switch to a very different kind of metagenome (we are currently looking at iHMP data for this).**

**This is the reason for our choice of approaches in Figure 2(a). For now, we think that an expanded analysis using real metagenomes is beyond the scope of the current paper, given length limitations.**

*Minor comment:

Figure 3(b) the y-axis should be "completeness" instead of "complete".*

**Fixed - thank you!**

Reviewer #2:

*Summary:
This manuscript details an efficient method by which metagenomic assembly graphs can be queried and explored to extract sequences and subgraphs of interest. This is an important contribution given that most metagenomic (and for that matter, genomic) assembly graphs are viewed as an intermediate step and are frequently discarded in favor of the (often heuristically) generated contigs that are computed by the various assembly algorithms. Information is inevitably lost by reducing an assembly graph to a set of (linear) contigs, as the graph structure is discarded when contigs are generated. In contrast, this manuscript convincingly argues that considering neighborhoods of the assembly graph can significantly improve not just metagenomic binning efforts but can also provide additional biological insight. For example, analyzing certain extracted neighborhoods can shed light on novel organisms in a metagenome, improve functional annotation of metagenomes, and illustrate the strain variation present in real-world samples. I applaud the authors for their work in effectively communicating the basic idea of dominating sets in Figure 1, as this figure elegantly summarizes the basic idea of the paper.

The main technical contribution that facilitates the exploration of assembly graph neighborhoods is the concept of a "dominating set": sets of nodes in a (compact) de Bruijn graph that are guaranteed to be within short graph distance to all nodes of the graph. This allows an index to be created that enables efficient queries and neighborhood extraction. The authors provide a proof that their linear-time dominating set generation algorithm returns a dominating set of size within a constant factor to the (NP-hard to calculate) optimal dominating set size. In practice, it appears that this algorithm is quite efficient, and results in an index that allows for fast queries (see Table 2) with total execution time under an hour.

In all, I believe this computational package, called spacegraphcats, will serve to further illuminate the complex metagenomic communities that are observed in the environment. The ability to efficiently extract and explore particular neighborhoods of an assembly graph will also allow researchers to focus on particular portions of the metagenome of interest without relying on a global assembly method that attempts to heuristically assemble an entire metagenome. The authors mention further interesting potential applications of this approach in the conclusion, and justifiably position this approach as an additional source of information when compared to sequence composition, contig abundance, and marker gene approaches. I believe this is an important contribution to the metagenomic literature, and potentially applicable to other fields as well (where large graph queries are relevant).*

**Thank you!**

*Software review:
I was able to get the software package installed (using Ubuntu) and the provided small test to run without any problem. Note that the line ``pip install https://github.com/dib-lab/khmer/archive/master.zip`` in https://github.com/spacegraphcats/spacegraphcats/blob/master/doc/installing-spacegraphcats.md is unnecessary as the requirements file https://github.com/spacegraphcats/spacegraphcats/blob/master/requirements.txt already contains the line ``https://github.com/dib-lab/khmer/archive/master.zip``*

**We have revamped the installation docs appropriately, thank you! Please see** [**https://github.com/spacegraphcats/spacegraphcats/pull/250**](https://github.com/spacegraphcats/spacegraphcats/pull/250)**.**

*I was also able to create my own workflow using an HMP mock community, and the software performed as expected (save for my initially overlooking the instruction to install BCALM 2; you may consider printing a message mentioning this given a standard user's penchant for copy-pasting install code from the *.md page).*

**The spacegraphcats workflow now emphasizes this through an error message, and the installation contains a test case that will flag the lack of BCALM. We also now suggest installing BCALM through conda (this is new: until a few months ago, the bioconda version of BCALM was broken).**

*There does remain, however, a few issues that could serve to improve the manuscript and facilitate its acceptance in the community.*

*Major comments:
I see three issues that may contribute to confusion to a reader of this manuscript, detailed below in descending order of importance.

First, while I understand that the target venue is not a mathematical/theoretical computer science journal, the details regarding the algorithms (and especially the proofs) assume quite a bit of background knowledge and make them somewhat difficult to parse. For example, the "distance-truncated transitive fraternal (dtf) augmentations" play a key role in Algorithm 1. However, the half-sentence description of what these are is insufficient to provide the reader with an understanding of the what a dtf augmentation actually does. The reference provided (24) is a 298 page thesis that provides little further understanding without significant investment of time on the part of the reader. Consider adding another sentence or two describing what these are, as the current description does not describe what the nodes and edges of the directed graph are that results from a dtf augmentation, nor makes clear whether $\omega(uv)$ is computed for all pairs of vertices $(u,v)$ or only for some. This also makes Algorithm 1 difficult to interpret as $\vec{G}_i \leftarrow \verb|dtfAugmentation|(\vec{G}_{i-1})$ leaves the reader wondering how the graph $\vec{G}_i$ has actually been transformed. Further issues in this regard include undefined notation: in Algorithm 2, the symbol $N(v)$ is used without any indication of what this is meant to represent. I image it's a neighborhood of sorts, but this should be made clear. This notation issue comes up again in the Appendix, where the claims and proofs are relatively straightforward to follow up until Lemma 3 where the mysterious symbol $\Delta^-(\cdot)$ is introduced. Not only is it not described what $\Delta^-$ is, but this is then applied to $\vec{G_{2r}} which is supposed to be a "2rth dtf-augmentation of $G$." Given none of these are defined or explained, it results in Lemma 3 being practically uninterpretable. Further uses of undefined terms include the repeated reference to graphs with "bounded expansion." From my understanding, bounded expansion is a property of a family of graphs, not a single graph. The manuscript does not make clear that compact de Bruijn graphs (as a family) have this property. Similarly, the claim near the end of Lemma 3 (page 10, line 32, column 2) makes the claim that their proof of a constant-factor approximation holds in graphs of bounded expansion without supporting this claim.*

**We expanded the explanation of the algorithm in the appendix. In particular, we added many definitions at the top of Appendix A and made sure that external references include the source’s numbering to make them locatable. In particular, the section now contains a self-contained explanation of how to compute dtf-augmentations.**

*Second, it may serve the reader well to have the following sentence near the front of the paper instead of relegated to the second to last paragraph of the conclusion "In this initial investigation of neighborhood indexing, we have focused on using neighborhood queries with a genome bin." As I read through the paper, I began to wonder why the authors were focusing so heavily on genome bins when I could imagine other relevant queries. This was not clarified (that this was a choice for the initial investigation) until the end of the paper. A reader may be well-served to understand that one can query with more than just a genome bin earlier in the manuscript. Along those lines, to further communicate how genome binning can be improved with neighborhood queries (though I understand this is by far not the only application of their method), the authors may consider using their approach (assemble, bin, query with bins, use extracted neighborhoods to assemble new bins) along with recent binning benchmarking software (https://academic.oup.com/gigascience/article/7/6/giy069/5034950) to show improvements beyond their analysis of completeness and gyrA analysis. Personally, I would be interested in seeing if other binning metrics (such as purity) are improved with the author's approach.*

**Thank you for the suggested reorganization! We’ve adjusted the last paragraph of the Introduction appropriately, and the relevant sentence now reads:**

**“Our initial investigations presented here focus on using this index to perform neighborhood queries in large assembly graphs to investigate genome binning and content recovery. This enables us to extract the genome of a novel bacterial species, recover missing sequence variation in amino acid sequences for genome bins, and identify missing content for metagenome-assembled genomes.”**

**Regarding binning evaluation, the lack of gold standard metagenome references is a challenge. For example, according to the instructions for the software described in the above paper, AMBER requires ““(1) a gold standard mapping of contigs or read IDs to underlying genomes of community members, … Benchmark metagenome sequence samples with a gold standard mapping can, e.g., be created with the CAMISIM metagenome simulator [4, 5]. A gold standard mapping can also be obtained for sequences (reads or contigs), provided that reference genomes are available, by aligning the sequences to these genomes.” For real metagenomes, we do not have gold standard reference genomes available. While we could benchmark spacegraphcats on synthetic metagenome data, we are concerned that synthetic metagenomes are not complex enough in their content (see the results in Fig 3(a) and Fig 4(a) in particular, which support this tentative claim).** *Lastly, the section on page 5 titled "Query neighborhoods assembled with Plass contain additional functional content" is difficult to follow. For example, "subtract(ing) any homolog already annotated in the genome bin" leaves the reader wondering where the homologs were subtracted from (from the possibly Plass assembled neighborhood? From the KEGG database? From the genome bin?). A bit more description about what is being performed here would be helpful.*

**We revised this to state, “We used KEGG to annotate the Plass-assembled amino acid sequences, and then removed any annotations already present for genes in the genome bin.”**

*Minor comments:

Page 1, Line 35 "genic content" is, in my experience, a bit non-standard, so consider replacing with "genomic" or "genetic" content depending on the intent.*

**We have replaced “genic” with “gene” content. Thank you!** *Figure 2: the colored lines in the left panel are not described. I assume they are linear regression lines, but it would be nice to have this explicitly stated.*

**Fixed, thank you!** *Figure 2: in the right panel, it is mentioned that "recovery is calculated as containment of target genome in query neighborhood." Is this meant to indicate containment on the basis of k-mers? If so, is this calculated in a brute force fashion? With a particular k-mer size? With sourmash and k=31 as briefly mentioned in the supplementary material? Furthermore, containment is not the only relevant metric here, as the neighborhoods extracted could be quite large (i.e. |neighborhood| - |target genome| is large even though |target genome \intersect neighborhood| / | target genome| is small). Perhaps mention the relative size of the neighborhood in comparison to the target genome (and describe if this is done at a particular k-mer size, etc.).*

**Yes, this was estimated with sourmash at a k-mer size of 31! We have updated the figure caption to make this explicit.**

**The question of containment vs similarity is of considerable concern for us. On mock or synthetic metagenomes, the Jaccard containment of the target by the neighborhood typically closely resembles the Jaccard similarity of the neighborhood with the target, because the neighborhood is usually quite small. This is probably because of a lack of strain variation in mock metagenomes.**

**On real metagenomes, however, similarity is often much lower than containment, because the neighborhoods are much larger (presumably because of strain variation!) We represent these much larger neighborhood sizes in Figure 3(a), and describe them in the paragraph starting with “We first ask whether genome query neighborhood sizes in a real metagenome differ from mock metagenomes.” (In some sense, the rest of the paper is about whether this additional content is real and meaningful).**

**We therefore chose to explicitly use containment because it properly distinguished between these two cases, and we feel that presenting the similarity numbers for the synthetic genome would be confusing to the reader. In particular, providing similarity numbers for Fig 2(a) would be misleading for real metagenomes, based on the results in Fig 3(a).**

**From a procedural/pipeline perspective, we are also focused on sensitivity more so than specificity, i.e. recovering a neighborhood that is inclusive but may be somewhat too large is a better error than recovering a neighborhood that is too small. Our position is that there are many tools to refine bins, but relatively few to expand them.**

*Page 4, line 51-52: I found the sentence "However, we found little improvement in the completion metrics" confusing as figure 3(b) appears to show significant improvement in the completion metric. Indeed, elsewhere in the paper the data in figure 3(b) is used to support the claim that "Neighborhood queries extend genome bins." Please clarify the contradiction that you observe "little improvement in the completion metric" and yet (page 6, lines 19-32) you find an "increase in the estimated completeness of most genome bins."*

**We agree this is confusing! The “little improvement” refers to the results of nucleotide assembly with MEGAHIT, and we believe that the amino acid assembly results with PLASS are a stronger demonstration of the value of neighborhood extension. We have adjusted the language on p4 to indicate “...we assembled reads from the query neighborhoods using MEGAHIT, and found this improved the completion metrics.”**

*Page 5, line 57 "ignoreed" typo*

**Fixed, thank you!**

*Page 5, line 48, column 2 "…useful to analyze sequence that has small graph distance…" consider "useful to analyze sequences that have small graph distance"*

**Fixed, thank you!**

*Page 7, lines 33-34, column 1 "…and then contracting long degree-two paths when possible." How does one contract a degree two path? Was a decision made to select one of the outgoing edges over another? This was unclear to me.*

**The contraction was done after removal of vertices of degree 1 (“spurs”), and is a second round of contraction (the first being done by BCALM). We have clarified the procedure as follows:**

**“..removing all vertices of degree one with a mean $k$-mer abundance of 1.1 or less. After the removal of these vertices, we then contracted any newly revealed degree-two paths.”**

**Here, contraction after spur removal can be done unambiguously in cases where there is a degree-two path, and no choice of edges is required.**

*Appendix A, page 11, line 15, column 1. There is no reference provided for BBHash. Consider providing one*

**Added! Thank you for catching this.**

**Second round of review**

**Reviewer 1**

I thank the authors for engaging with my comments and attempting to address the points I made previously.
I do not have any additional comments.

**Reviewer 2**

The authors have thoroughly addressed the issues I raised in my initial review. In particular, I appreciate their expansion of the technical details in the Appendix, as it is now much easier to understand Algorithms 1 and 2 and the approximation guarantee.

As for the binning evaluation, I understand the concerns about simulated metagenomes not being complex enough in their content, and agree that space constraints preclude such an inclusion in this manuscript. Even so, it would be interesting (from a methods developer perspective anyway) to see how spacegraphcats might be used as (or improve existing) binning techniques in some future work.

I also appreciate the clarification about the focus on sensitivity more than specificity when recovering neighborhoods as this clarified the choice of metrics the authors chose in Figures 2 and 3.

Quibbling typo: Page 10, column 1, line 48 "to which graph of bounded maximum degree..." -> "to which graphs of bounded maximum degree"
